# Computational imaging reveals mitochondrial morphology as a biomarker of cancer phenotype and drug response

**DOI:** 10.1038/srep32985

**Published:** 2016-09-09

**Authors:** Randy J. Giedt, Paolo Fumene Feruglio, Divya Pathania, Katherine S. Yang, Aoife Kilcoyne, Claudio Vinegoni, Timothy J. Mitchison, Ralph Weissleder

**Affiliations:** 1 Center for Systems Biology, Massachusetts General Hospital, Harvard Medical School, 185 Cambridge St., CPZN 5206, Boston, MA 02114, USA; 2 Department of Neurosciences, Biomedicine and Movement Sciences, University of Verona, Strada Le Grazie 8, 37134 Verona, Italy; 3 Department of Systems Biology, Harvard Medical School, 200 Longwood Ave, Boston, MA 02115, USA

## Abstract

Mitochondria, which are essential organelles in resting and replicating cells, can vary in number, mass and shape. Past research has primarily focused on short-term molecular mechanisms underlying fission/fusion. Less is known about longer-term mitochondrial behavior such as the overall makeup of cell populations’ morphological patterns and whether these patterns can be used as biomarkers of drug response in human cells. We developed an image-based analytical technique to phenotype mitochondrial morphology in different cancers, including cancer cell lines and patient-derived cancer cells. We demonstrate that (i) cancer cells of different origins, including patient-derived xenografts, express highly diverse mitochondrial phenotypes; (ii) a given phenotype is characteristic of a cell population and fairly constant over time; (iii) mitochondrial patterns correlate with cell metabolic measurements and (iv) therapeutic interventions can alter mitochondrial phenotypes in drug-sensitive cancers as measured in pre- versus post-treatment fine needle aspirates in mice. These observations shed light on the role of mitochondrial dynamics in the biology and drug response of cancer cells. On the basis of these findings, we propose that image-based mitochondrial phenotyping can provide biomarkers for assessing cancer phenotype and drug response.

Mitochondria are involved in a variety of cellular functions, including ATP production, amino acid and lipid biogenesis, signaling and apoptosis^1^,^2^,^3^. The number and morphology of mitochondria within a given cell (i.e. a cell’s mitochondrial phenotype) vary with cell type, differentiation stage, energy requirements, overall cell health and cell cycle^4^. Mitochondrial dysfunction has been widely linked to cancer, aging and degenerative diseases^5^. Recent work has elucidated some of the molecular mechanisms that regulate mitochondrial abundance, morphology^6^ and function^7^. For example, we know from work in cell lines that mitochondria can switch between a fragmented phenotype, with ovoid-shaped mitochondria, and a reticulum, with a complex branched structure^8^,^9^. Indeed, the morphology of mitochondria in the past has been classified by multiple methods, the most simple of which has been to divide individual mitochondria into short, fragmented mitochondria, termed punctate, intermediately sized mitochondria, and elongated, highly branched mitochondria termed filamentous^10^,^11^,^12^,^13^,^14^. Mechanistic studies of mitochondrial dynamics in cultured cells have shown that mitochondrial fission and fusion are mediated by post-translational modifications in key proteins including Drp1/Fis1 and Mfn1&2/Opa1, respectively^15^. Additionally, recent work has illustrated that highly networked mitochondria often cluster in perinuclear regions and interact with the endoplasmic reticulum^16^. These studies have revealed mechanisms that control mitochondrial morphology but the relationship between cellular states and mitochondrial morphology is still poorly understood.

The dynamic nature of mitochondria, and potential mechanistic connections between their morphology and cell state (Fig. S1), suggest that mitochondrial phenotype might provide a biomarker for cancer diagnosis and/or treatment. Despite this potential utility, mitochondrial phenotype, particularly at the cell population level, has been studied less extensively. Current knowledge mostly relies on stained sections^17^ or cell culture^18^ that illustrate individual mitochondrial morphologies ranging from filamentous to punctate, yet no comprehensive analysis has been conducted to date quantifying cellular mitochondrial phenotypes. Intrigued by the highly dynamic appearance and rapid morphological changes in mitochondrial patterns (MovieS1), we set out to profile them more comprehensively in cancer cells. We were particularly interested in determining the structural differences in cell populations, between different cancer types, over time and in freshly harvested patient samples. We argued that cellular mitochondrial phenotype profiles could ultimately be used as a biomarker of a cell’s metabolic state and efficacy of anti-proliferative therapeutic intervention. In order to analyze mitochondrial phenotype reliably, it was necessary to develop an analytical platform which minimized artifacts due to cell damage during cell isolation and fixation.

Biomarkers, defined as a characteristic that is “objectively measured and evaluated as an indicator of pathogenic processes or pharmacologic responses to a therapeutic intervention”^19^ have become essential tools as primary endpoints in clinical trials. Once validated, a biomarker can decrease the cost of trials and predict outcomes earlier. However, there remains a clinical need to more effectively measure cellular phenotypes in clinical samples. Currently, harvested cells and tissues are often collected at two ends of a spectrum: (i) genomic analyses which reveal driver oncogenes and specific mutations^20^,^21^ and (ii) protein analyses of handpicked biomarkers monitor cellular response^22^,^23^. Ideally, clinical samples are collected serially to monitor change in key protein expression levels. This raises many challenges, notably morbidity risk of repeat core biopsies, high cost and logistical limitations. Alternative sample collection methods include fine needle aspirates (FNA), or analysis of rare cells present in other easily accessible fluids. However, these samples have much lower cell numbers than biopsies, thereby limiting the number of molecular analyses. Morphologic phenotyping of mitochondrial states in few scant cells such as proposed here, in addition to providing valuable biological information, could thus fill a clinical need.

## Results

### Analytical pipeline to assess mitochondrial phenotype

**Figure 1:**
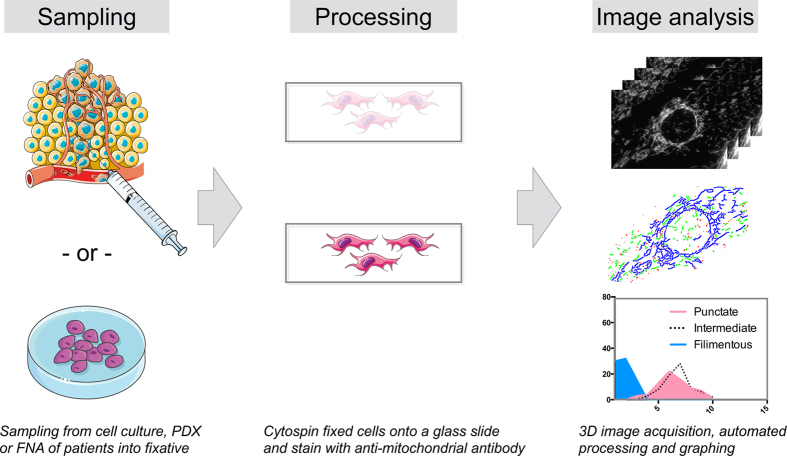
Workflow for processing cell samples. Cells are sampled by fine needle aspiration of intact tumors or from culture. Harvested cells are immediately fixed and stained. Images of individual cells (usually ~100 cells per sample) are captured and processed by an image analysis, segmentation and classification algorithm (See Figs S3 and S4, for details). Artwork utilized from the open source public database Servier Medical Art (http://www.servier.com/Powerpoint-image-bank), under a Creative Commons Attribution 3.0 Unported License.

**Figure 2:**
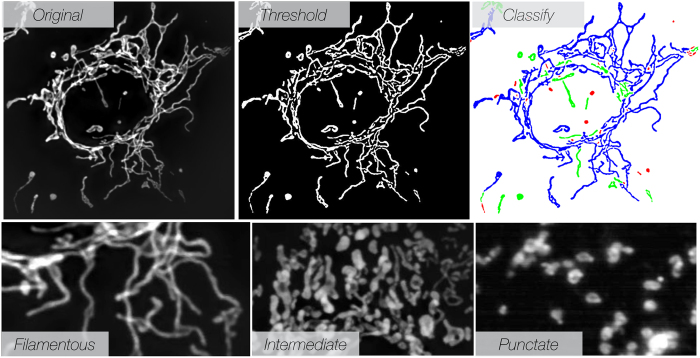
Representative examples of mitochondrial segmentation and classification. An original stack of mitochondria following 3-D filtering and reconstruction is mapped into a single gray scale image(top left panel). The resulting image is then thresholded (top middle panel), and mitochondria are automatically classified (top right panel) into one of three mitochondrial types (bottom panels). See Figs S3 and S4 for details on the classification algorithm.

Our initial goal was to develop a processing pipeline to reliably quantify mitochondrial phenotype in both human biopsies and cultured cells. Individual mitochondrial morphology is highly dynamic over time, as revealed by live imaging of Mito-GFP cells (Movie S1). Using live cell imaging as a gold standard to define mitochondrial phenotype, we set out to develop a more widely applicable method for fixing, imaging and analyzing freshly harvested cells such as biopsy samples (Fig. 1). Conventional fixation methods such as methanol, lyse/fix buffers and rapid freezing resulted in destruction of mitochondrial networks and non-representative samples (Fig. S2). Interestingly, this may explain the often divergent results published in the literature^18^,^24^. In the optimized method, we used a cytoskeletal buffer^25^/4% paraformaldehyde (PFA) solution as a fixative. We placed this buffer in a syringe to harvest cells from primary tumors via fine needle aspirates or as a typical immunocytochemistry fixative for cell culture samples. After fixing for 10 minutes, we stained the cells on glass slides, and acquired Z-stacks on either fluorescence or confocal microscopy systems. We processed cellular Z-stacks with a unique filtering and analysis algorithm (Fig. 2). The novelty of this method is the utilization of a 3-D all-in-focus algorithm^26^ to combine multiple Z-layers of mitochondria into a single image (Fig. S3). Mitochondria in this image were then segmented and classified using the random forest computer learning algorithm to place identified individual mitochondria into a simplified scheme of mitochondrial groupings (punctate, intermediate or filamentous) based on a variety of shape descriptors for each object (Figs S4 and S5). This algorithm was tested in several ways. Firstly, we created an in silico data set with artificially created mitochondria which a human would describe as either punctate, intermediate or filamentous. These artificial mitochondria were then subjected to the classification algorithm, where each individual set of mitochondria were accurately classified. Secondly, using a training set of 200 actual mitochondria found in our images following application of the Z-stack algorithm, we compared manual classification by a human to the classification produced by our algorithm and found less than 4% error. We additionally analyzed OVCA-429 cells either not treated or treated with known fragmentation agents (FCCP and Antimycin A) to check algorithm performance, verifying that mitochondrial fragmentation was detected using these agents (Fig. S6). Following analysis, we evaluated data on a single-cell basis across cell populations.

### Measuring Mitochondrial Heterogeneity in Cultured Human Cancer Cells

**Figure 3:**
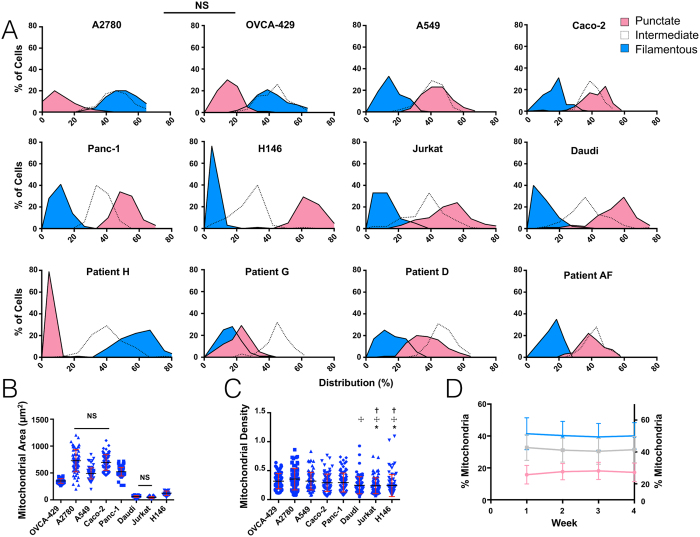
Population level mitochondrial heterogeneity. (**A**) Adherent and non-adherent cultured cell lines (top two rows) or patient derived xenograft samples (third row) were fixed, stained and imaged. 100 randomly sampled single cells from each cell type were analyzed and their respective mitochondria classified. Data are shown as percentage of mitochondria per cell present in each respective classification for each cell line. (**B**) The total mitochondrial area (NS = Not significant at p < 0.01) and (**C**) density of mitochondria for cultured cell lines was also examined (*Significant from OVCA-429 cells, Significant from A2780 cells, ^†^Significant from A549 cells at p < 0.05). (**D**) To determine whether a given phenotype pattern changes over time, OVCA-429 cells were repeatedly analyzed over a 4 week period. Note the relative stability in population patterns. Left Y-axis represents the percentage of mitochondria for punctate (red) and filamentous (blue) mitochondria while right axis represents the percentage of mitochondria for intermediate (grey) mitochondria. No weekly statistical differences were found at p < 0.01.

Figure 2 shows an example of image acquisition, thresholding and automated image analysis. We routinely acquired and analyzed ~100 cells per experiment (see Fig. S6 for an output example). Single cell-level acquisition enabled analysis of mitochondrial morphological phenotypic heterogeneity both within same-type cell populations as well as among different cell types and tissues. To profile mitochondrial phenotypes, we first focused on the ovarian cancer cell line OVCA-429. In this cell line, ~40% of mitochondria were filamentous, ~40% were punctate and the remainder showed an intermediate phenotype. These proportions varied across different cell types. Figure S7 presents the plots of 100 analyzed cells showing typical distribution width. The total mitochondrial area was 400 μm^2^, and the density was 0.33 ± 0.16 (mitochondrial area/total cellular area). Interestingly, the fraction of different mitochondria populations, sizes and densities in both OVCA-429 cells and other profiled populations remained remarkably constant over time, at least during the four weeks of observations recorded in this study (Fig. 3D). This finding provides further evidence that mitochondrial patterns, while stochastic, are pre-programmed as a whole.

To determine mitochondrial phenotypes among different tumors, we next analyzed adherent (A2780, A549, Caco-2, OVCA-429, Panc-1) and non-adherent cell lines (Daudi, Jurkat and H146) as well as low passage, patient-derived cells from ovarian cancer patients AF, D, G and H. The mitochondrial phenotype varied from highly filamentous with few punctate mitochondria (e.g. OVCA-429 and Patient H) to completely fragmented, a phenotype present in both adherent and non-adherent cell lines (Figs 3A and S7–S9). Interestingly, there was considerable diversity among patients, even in ovarian cancer cells obtained and cultured under identical conditions. Accounting for different cell sizes, we also analyzed the density of mitochondria (mitochondrial area divided by total cell size), a result which demonstrated a much higher degree of uniformity.

### Correlations between mitochondrial phenotype and bioenergetics

**Figure 4:**
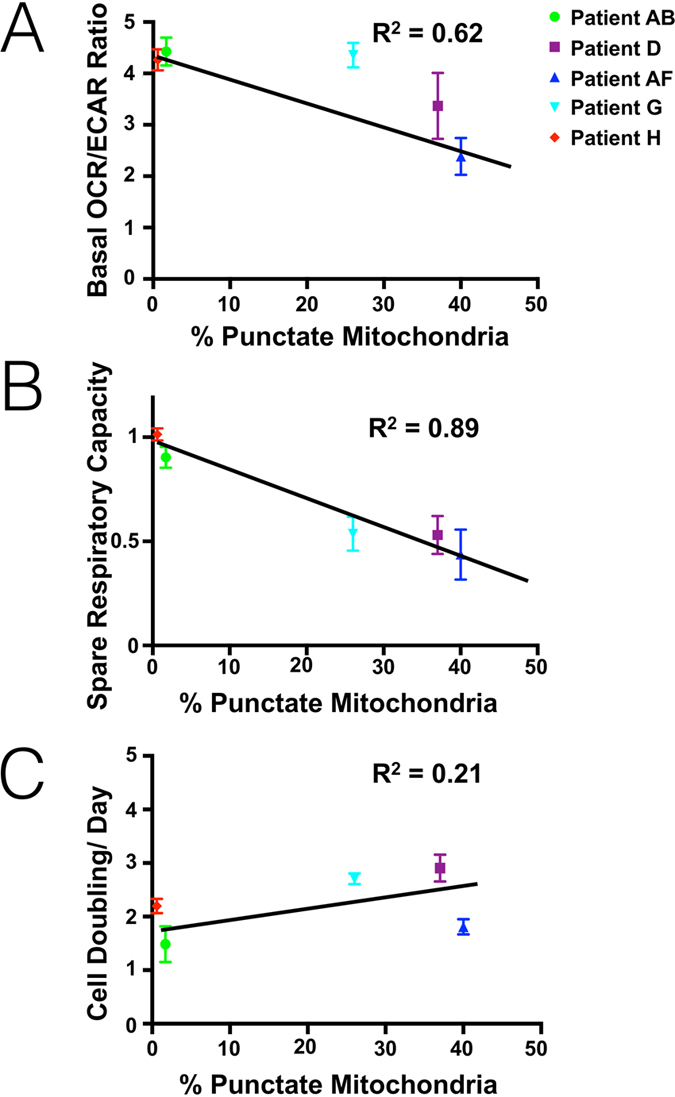
Metabolic parameters in patient derived xenograft models. Cells from the five patient derived xenograft models were analyzed for mitochondrial phenotype and processed in parallel using a Seahorse bioenergetics analyzer. Note the correlation between basal oxygen consumption rate/extracellular flux rate (**A**) and spare respiratory capacity (**B**) as a function of punctate phenotype. In contrast, there was only a weak correlation between punctate mitochondria and cell doubling rate (**C**).

To understand the relationship between mitochondrial phenotype and overall cellular metabolism, we measured our patient-derived samples’ spare respiratory capacity, basal oxygen consumption rate (OCR, a measure of oxidative phosphorylation) and basal extracellular acidification rate (ECAR, a measure of glycolysis). As summarized in Fig. 4, there was an inverse relationship between OCR/ECAR rates and the amount of punctate mitochondria in these samples, which were cultured under identical conditions. In other words, punctate mitochondria correlated with increased glycolysis levels and decreased oxygen consumption (Fig. 4A). Similar observations held true for spare respiratory capacity, which was lower when more punctate mitochondria were present (Fig. 4B). Additionally, we saw no correlation between mitochondrial phenotype and cellular grown rates in patient-derived samples (Fig. 4C). Measurements of common mitochondrial morphology protein levels showed limited correlation with total protein levels (Fig. S10).

### Mitochondrial morphology is an early biomarker of therapeutic intervention

**Figure 5:**
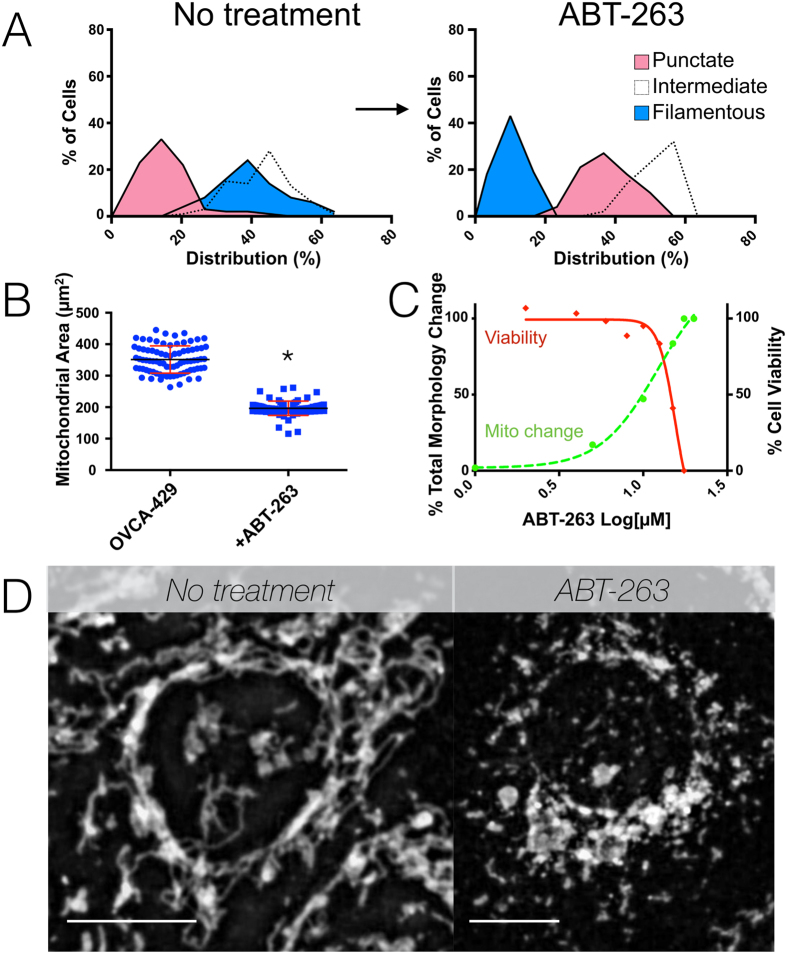
Effect of pro-apoptotic treatment on mitochondrial morphology and heterogeneity. (**A**) OVCA-429 cells were analyzed either without or 72 hours after treatment with 10 μM ABT-263 (navitoclax). Note the considerable reduction in both filamentous mitochondria and total mitochondrial area (**B**). (**C**) Dose response curve of ABT-263 plotting cell viability(solid red line, right axis) or the change in mitochondrial morphology defined as the sum of the percentage change from pre to post-treatment for each of filamentous, intermediate, and punctate mitochondria) (dashed green line, left axis) with treatment. Mitochondrial changes occur earlier and in lower doses compared to cell viability measurements. (**D**) Representative images of control (left panel) and treated (right panel) OVCA-429 cells. Scale bars represent 5 μm.

We suspected that anti-proliferative and targeted pro-apoptotic treatments would affect mitochondrial morphology (possibly through indirect or stress mediated pathways), but exactly which changes would be most prevalent remained unclear. We therefore chose to utilize two drugs, ABT-263, a mitochondrial pathway of apoptosis targeted treatment, and cisplatin, a common clinical chemotherapy treatment, as test cases to examine mitochondrial phenotypic heterogeneity. To examine mitochondrial morphology we used a mitochondrial GFP reporter to transduce OVCA-429 cells. As expected, ABT-263 treatment resulted in a dose-dependent increase in punctate mitochondria (Fig. 5). Interestingly, these phenotypic changes occurred earlier than changes in cell viability. We further used cisplatin to perform similar measurement in which treated OVCA-429 cells showed alterations in mitochondrial morphology and mass, and exhibited morphological changes at lower doses than those which caused cell death as well (Fig. S11). In both of these treatments, the heterogeneity of mitochondrial morphology present in pre-treated samples was retained following treatment with the medians of the curves shifted toward a more punctate phenotype. Thus, mitochondrial morphology appears to be a sensitive indication of drug action for at least some classes of drugs, which precedes cell death. Further mechanistic study of possible changes in proteins will be needed to confirm this finding for individual drug classes.

### Fine needle aspirations of tumors allow therapeutic efficacy measurements

**Figure 6:**
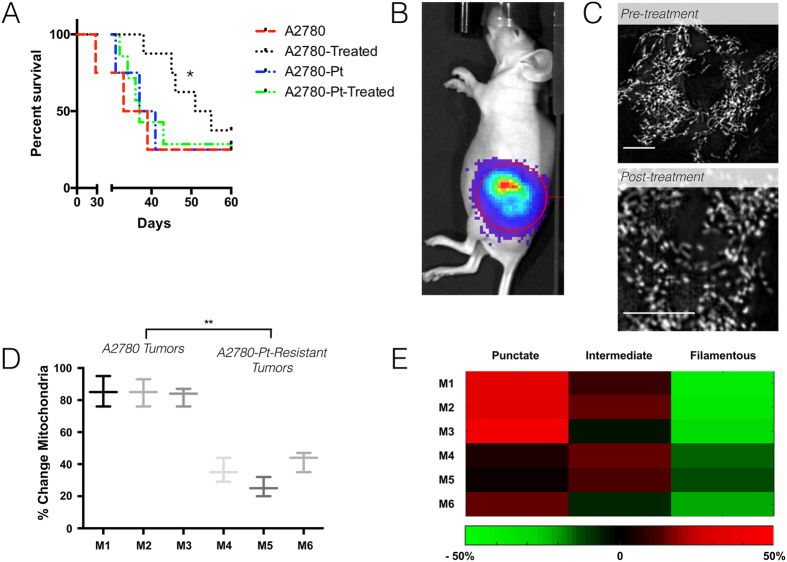
Mitochondrial morphology as a biomarker to assess drug treatment. Mice (Marked in the figure as M1, M2 etc.) were implanted with A2780 (PT sensitive) or A2780-pt resistant tumors, and fine needle aspirates were taken both before and after cisplatin treatment. (**A**) Kaplan Meier curves for mice inoculated with A2780 and A2780-Cisplatin resistant tumors, either treated or not treated with cisplatin therapy. Groups consisted of 7 mice each. *Significant at p < 0.05. (**B**) Example mouse with A2780-cisplatin resistant tumor expressing luciferase used to confirm tumor growth. (**C**) Examples from cells both pre-treatment (top) and post-treatment (bottom) following fine needle aspiration are presented. Scale bars represent 5 μm. (**D**) Results from several treated animals show a representative pattern. (**D**) Summarized analysis of mitochondria from 50–100 cells taken from each of 3 representative mice inoculated with A2780 and A2780-Pt tumor types and treated with cisplatin. The % of mitochondrial change was again calculated as the sum of the percentage change from pre to post-treatment for each of filamentous, intermediate, and punctate mitochondria). (**E**) Heat map of the percentage change pre- and post-treatment for each mitochondrial type for 3 representative mice from either treated A2780 or A2780-Pt mice. **p < 0.01.

Based on the *in vitro* results (Fig. S11), we hypothesized that mitochondrial phenotype might serve as a biomarker of chemotherapy response *in vivo*. We prepared xenografts of murine intrabursal implanted tumors using the well-established model^27^ of A2780 and A2780 platinum-resistant human ovarian cancer tumors. We then collected representative fine needle aspirates before and after delivering a first dose of cisplatin chemotherapy to the mice. Therapy in mice was continued to generate Kaplan-Meier curves. Mitochondrial phenotype was slightly more fragmented in biopsies than in samples from the two cell lines grown in cell culture. In samples drawn following cisplatin treatment, A2780 cells showed considerable mitochondrial fragmentation (albeit with greater heterogeneity than *in vitro* samples), while A2780 platinum-resistant cells phenotype retained a phenotype similar to their pre-treatment controls (Figs 6 and S12).

## Materials and Methods

### Cell Culture and Treatment

Cell lines were selected to give a broad overview of multiple cancer types, including breast, ovarian, pancreatic, and hematologic cancers. All cell lines except A2780, A2780-cis and patient derived lines were obtained from ATCC and cultured as directed. Patient-derived cell lines were obtained and cultured as previously described^28^. Briefly, patient-derived cells were obtained from ovarian cancer patients and were cultured under identical conditions as described^28^. The A2780 and cisplatin-resistant A2780 cell lines were obtained from Sigma-Aldrich. These cell lines were transduced with a mitochondrial targeted-GFP using a lentiviral construct consisting of mito-PAGFP (Addgene Plasmid #23348), where the mitochondrial targeting sequence and GFP were cut and inserted into a pLVX-AcGFP1-C1 Vector backbone (Clonetech #632155). Lentivirus production was completed following a standard protocol^29^. Cisplatin and ABT-263 were obtained from Sigma-Aldrich and dissolved in DMF and DMSO, respectively, prior to cell treatment.

### Cell Fixing and Immunocytochemistry

Cells were fixed via incubation for 10 minutes in a solution consisting of 4% Paraformaldehyde (Electron Microscopy Sciences) dissolved in Cytoskeletal Buffer^25^. Adherent cells were fixed by removing cell media and immediately adding fixation solution. Non-adherent cells were taken directly from cell culture and immediately placed in PFA fixative; following fixation, cells were prepared in a thin layer on a slide using a Cytospin system (Thermo Scientific). Fixed cells were then subjected to typical immunofluorescence staining. Briefly, following a spin-down period, cells were washed 3X in TBS and permeabilized in 0.1% Triton X in TBS for 30 minutes at RT. Cells were then blocked for an additional 30 minutes using Odyssey blocking buffer (LI-COR Biosciences) at RT according to the manufacturers’ directions. Following blocking, slides were incubated with TOMM-20 (Abcam #ab56783) antibody at a concentration of 3 μg/mL for 1 hour at RT, washed 3X in TBS and incubated for 1 hour with a secondary antibody (Alexa Fluor 594) at RT. After washing 3X in TBS, slides were mounted with Vectashield anti-fade medium with DAPI (Vector Laboratories) and sealed with a coverslip for imaging.

### Image collection

Images were collected as a Z-series using a BX-63 Upright Automated Fluorescence Microscope. Images were taken in the Z-direction every ~0.5 μm for variable ranges depending on the height of the cell line being imaged on a 100X oil objective at a resolution of 2560 × 2160 pixels using Metamorph Software. For each cell line, sufficient images were taken to ensure that there were ~100 usable individual cells for follow-up analysis. Individual cells were counted via DAPI staining.

### Image Analysis

Images were analyzed using Matlab scripts developed in house. Individual cells within each image were first manually selected based on nuclear DAPI staining. Following individual cell selection, mitochondria were thresholded using a multi-step process. Briefly, Z-stacks of cells were first compiled into a single image based on an all-in-focus algorithm^26^. From this single image, “rolling ball” background subtraction was conducted with a ~50 pixel averaging subtraction ball. Images were then run through a fast Fourier transform-based band pass filter and thresholded using an adaptive thresholding algorithm^30^. From this image, basic open-close morphological operations eliminated noise generated during processing to create a final binary image displaying regions identified as mitochondria. Indexed shape properties of each region were then logged, and individual mitochondria were classified into one of three categories; punctate, intermediate or filamentous. These 3 categories were chosen on the basis of previous literature classifications^10^,^11^,^13^,^14^. Classification was completed automatically using the random forest algorithm included in Matlab combined with a training set of ~1000 manually classified mitochondria. Total mitochondrial morphology change was calculated by adding the percentage change in morphology for each category (i.e. change from initial condition or individual category). All data was compiled and analyzed in either Prism or Matlab.

### Oxygen Consumption and Extracellular Acidification

Oxygen Consumption Rate (OCR) and Extracellular Acidification Rate were measured using an XFe96 Extracellular Flux Analyzer (Seahorse Bioscience). For each cell line, cells were seeded in several concentrations, ranging from 15,000 cells/well to 25,000 cells/well. Prior to experiments, wells were washed and resuspended in Seahorse XF Base Medium supplemented with 1.0 mM sodium pyruvate and 10.0 mM glucose. To calculate mitochondrial parameters, cells were treated with 0.5 μM oligomycin A followed by a range of FCCP (0.5, 1 or 2 μM concentrations) and, finally, a mixture of 1 μM antimycin A and 1 μM rotenone. Oxygen consumption and extracellular flux rate were measured in triplicate over 10 minutes at the beginning of each experiment and following the addition of each respective compound. Values were calculated from curves consistent with manufacturer instructions.

### Animal Models and Fine Needle Aspiration

All animal experiments were carried out in accordance with guidelines from the Massachusetts General Hospital Institutional Subcommittee on Research Animal Care. Experimental protocols were approved by the Massachusetts General Hospital Institutional Subcommittee on Research Animal Care under Protocol #2015N000157. Nude mice (Cox7, Massachusetts General Hospital) received an intrabursal injection of approximately 3–4 × 10^6^ cells suspended in 1:1 PBS of either A2780 or A2780-cisplatin resistant cells expressing mitochondrial-targeted GFP and allowed to grow for ~4 weeks. When the tumors became palpable, mice were anesthetized with 2% isoflurane in 2 L*min^−1^ oxygen on a heated microscope stage. Fine needle aspiration (3 passes) of the tumor area was conducted via a 10 mL syringe preloaded with the described fixation buffer. The following day, mice were again anesthetized and then injected via tail vein catheter with a previously established maximum tolerated dose of 10 mg/kg cisplatin dissolved in 0.1% NaCl. In the same procedure mice were hydrated with 100 μL of saline solution via IP injection. After 24 hours, fine needle aspiration was again performed on the mice as described above.

After fine needle aspirates were acquired in fixative, cells were incubated for another 10 minutes. Cells were then spun down, and fixed erythrocytes were lysed by treatment in 0.1% Triton X as previously described^31^. Following lysing, cells were resuspended in 100 μL of PBS and placed on a slide via cytospin. Slides were then washed 3X in TBS, mounted with Vectashield anti-fade medium with DAPI (Vector Laboratories) and sealed with a coverslip for imaging.

### Cell Viability Assays

Cell viability assays were conducted by dosing cells with gradient concentrations of drugs, including ABT-263 and cisplatin, using an HP D300 Digital Dispenser. Following 72 hours incubation, cells were incubated with Presto Blue Cell Viability reagent in accordance with the manufacturer’s recommendations. Results were read on a TECAN fluorescent plate reader and were normalized to highest and lowest control values for data presentation and analysis.

### Western blot

Cells were grown to confluence, washed twice with ice-cold PBS and then lysed in radioimmunoprecipitation buffer (RIPA, 89900, Pierce) containing HALT protease inhibitor cocktail (87786, Pierce). Lysates were transferred to microfuge tubes, incubated five minutes on ice, and then centrifuged at 14,000 × g for 15 min at 4 °C to remove cellular debris. Total protein was measured using the BCA assay (23227, Pierce) and equal protein was loaded on a 4–12% NuPAGE Bis-Tris gel (NP0322BOX, Life Technologies). Protein was then transferred to nitrocellulose, blocked in SuperBlock T20 (TBS) (37536, Pierce), followed by brief washing in TBS containing 0.1% Tween-20 (TBST). Blots were incubated overnight at 4 °C in Drp1 (8570, Cell Signaling Technology), Mfn1 (14739, Cell Signaling Technology), Mfn2 (11925, Cell Signaling Technology) or Opa1 (67589, Cell Signaling Technology) primary antibody diluted 1:1000 in SuperBlock. Blots were washed three times, 5 min each, followed by a one hour incubation in HRP-conjugated secondary antibody. Following secondary incubation, blots were again washed three times, 5 min each in TBST followed by detection using SuperSignal West Pico chemiluminescent substrate (34077, Pierce). Expression was quantified by densitometry using ImageJ (NIH) and evaluated both normalized to GAPDH expression and independently (data not shown).

### Statistical Analysis

For mitochondrial distribution analysis, we compiled values for three separate experiments, each of which included ~50–100 cells. Our analysis used Friedman’s rank sums test to identify any statistical differences among the distributions. For sets in which a difference was detected, Dunn’s post-hoc test was used to determine group subset differences. The *p* values were considered significant when less than 0.01. Populations were statistically significant from each other unless otherwise noted. Multiple comparison tests were conducted via ANOVA with Tukey’s test with all populations compared to each other. Weekly measures of mitochondrial morphology were analyzed via repeated measures ANOVA. For these tests, NS = Non-Significant at specified p values. Significance of Kaplain-Meir curves was determined by conducting multiple comparisons of curves via log-rank test. For correlations, the Pearson product-moment correlation coefficient was calculated and noted on plots in all cases.

## Discussion

To date, our understanding of mitochondrial morphology in cancer has derived primarily from electron microscopy images taken from preserved sections of tumors or cells. While these studies of mitochondria have yielded important information regarding alterations in individual mitochondrion ultrastructure as well as the molecular components controlling mitochondrial morphology, imaging at this scale does not allow for larger-scale analysis of the whole cell mitochondrial network phenotype at the single cell and cell population levels. Here we have developed such a methodology for analyzing mitochondrial morphology. While previous methods have been utilized to automatically classify mitochondria into various groupings^13^,^32^,^33^, our work differs in our use of a 3-D all in focus algorithm, which allows us to directly compare optically diverse cell types and automatically classify them in a manner consistent with previous work. Using this method of 3-D reconstruction and semi-automated analysis of immunofluorescent images, we can therefore analyze and qualitatively compare the heterogeneity of mitochondrial morphology within individual cell lines and among different cell populations in large numbers of cells. Thus, for the first time we are able to quantitatively understand mitochondrial phenotypic heterogeneity and how it is altered between different cancer types as well as from drug treatments.

Our analysis of mitochondrial phenotype revealed broad variation both within cell lines and among different cell populations. This heterogeneity ranged from cells with almost completely punctate mitochondrial phenotypes to cells with almost completely filamentous phenotypes. Similarly, analyzing patient-derived ovarian cancer cells, notably cultured under identical conditions eliminating the possibility of cell culture factors being the primary source of heterogeneity, yielded results with a high level of phenotypic diversity from patient to patient. Taken together with data showing unaltered mitochondrial phenotype during weekly checkpoints under cell culture, we conclude that while mitochondrial morphology and phenotype at the single mitochondria and single cell level is subject to high levels of change and remodeling, mitochondrial phenotype is stable in cell populations. Furthermore, this characteristic stability is highly heterogeneous among cell lines and individual patient tumors.

We hypothesized that mitochondrial phenotypic differences among cell lines would correlate with changes in cell metabolism. Our findings show that in general, shorter and more punctate mitochondria correlate with (a) tumor cells’ preference to use glycolysis over oxidative phosphorylation and (b) less functional mitochondria in our patient samples. This finding is significant for several reasons. Firstly, there is a link between more aggressive tumor phenotypes and increased glycolysis levels^34^. More recent evidence shows that targetable factors such as HIF-1a and PI3K result in higher glycolysis levels^35^,^36^. While here we illustrate correlative studies of mitochondrial morphology and metabolism we hypothesize that heterogeneous mitochondrial morphology may represent a readily attainable marker of tumor metabolic needs and, potentially, overall patient prognosis. Follow on work will be needed to confirm such hypotheses at the molecular level. Additionally, the proteins controlling mitochondrial phenotype interact with a large and steadily increasing number of proteins with important roles in cell health, adaptability and survival. Indeed, analysis of mitochondrial morphology proteins including Drp1, Mfn1/2 and Opa1 showed only limited correlation with overall mitochondrial morphology. Taken together, these findings illustrate the multifaceted nature of determining mitochondrial phenotype and its possible unique role as a broad indicator of cell condition and response, or not, to therapy. Additional study will be required to completely understand the factors controlling mitochondrial morphology and its response to drug treatment.

Previous literature has noted that mitochondrial morphology is altered *in vitro* by chemotherapy^37^,^38^. Indeed, when we treated OVCA-429 cells with the pan Bcl-2 inhibitor ABT-263, at drug dosing levels that precluded the possibility of changes being associated with apoptosis initiation alone, we observed increased fragmentation *in vitro*. Using a first line chemotherapy treatment, cisplatin, we observed similar changes in overall mitochondrial phenotype, including increased fragmentation/the number of punctate mitochondria, as well as an increase in the average size of elongated mitochondria in the case of cisplatin. In both the case of ABT-263 and cisplatin, we observed highly heterogeneous responses to the drug treatments, illustrating the need for population level analyses as completed here when analyzing the effects of drugs on mitochondrial morphology, as such heterogeneity may be significant in determining overall tumor response to treatment. Next, we illustrated that it is possible to observe mitochondrial phenotype in cells derived from fine needle aspirates commonly conducted in the clinic. We observed that cells collected in this manner had somewhat more punctate mitochondrial phenotype than cells observed *in vitro*. This result may indicate that mitochondrial phenotype *in vivo* differs from what is observed in cell culture. We also documented mitochondrial phenotype alterations caused by drug treatments, thereby indicating the clinical relevance of analyzing mitochondrial morphology. In these samples, the presence of heterogeneous mitochondrial phenotypes indicates the still functional state of at least a subset of cells, illustrating that apoptosis alone is not the sole cause of mitochondrial morphological changes. Going forward, this method of observing mitochondrial phenotype when combined with additional protein based analyses and follow on studies could prove invaluable as an early evaluation of patient response to treatment and a warning of developing resistance to chemotherapy. A follow-up study will examine the prognostic value of mitochondrial phenotype via fine needle aspirate in a variety of patient tumor types, as this phenotype appears to correlate closely with metabolic alterations in cells and may therefore reveal targetable susceptibilities with follow-on protein analyses.

## Additional Information

**How to cite this article**: Giedt, R. J. *et al*. Computational imaging reveals mitochondrial morphology as a biomarker of cancer phenotype and drug response. *Sci. Rep.* **6**, 32985; doi: 10.1038/srep32985 (2016).

## Supplementary Material

Supplementary Information

Supplementary Movie S1
