# Supplementary material for: Computational imaging reveals mitochondrial morphology as a biomarker of cancer phenotype and drug response
Source: Sci Rep. 2016 Sep 9;6:32985. doi: 10.1038/srep32985 (PMC5017129; doi:10.1038/srep32985)
Supplement: Supplementary Information [file srep32985-s1.pdf]

Supplementary Information

# Computational imaging reveals mitochondrial morphology as a biomarker of cancer phenotype and drug response

Randy J Giedt[1], Paolo Fumene Feruglio[1,2], Divya Pathania[1], Katherine S Yang[1], Aoife Kilcoyne[1], Claudio Vinegoni[1], Timothy J Mitchison[3,], Ralph Weissleder[1,3,*]

Movie S1: Short term movement of mitochondria. OVCA-429 cells expressing mitochondrial GFP were imaged at 1 frame per second for 20 minutes.

Figure S1. Illustration of the multifaceted factors which control mitochondrial phenotype. Past research has shown that mitochondrial phenotype is altered by almost all factors in which mitochondria play a role in the cell. We therefore hypothesized that utilizing mitochondrial phenotype and its' change as a biomarker in cancer would yield a novel, highly relevant indicator of tumor condition and response, or not, to therapy.

Figure S2. Examples of fixation techniques and their effects on mitochondrial morphology. OVCA-429 cells were fixed by 4% paraformaldehyde (PFA) in cytoskeletal buffer (CSB) as described in the methods section, ice cold methanol or Lyse/ Fix buffer (BD Phosflow). In the case of methanol and lyse/ fix buffer, high amounts of mitochondrial fragmentation were observed, leading to the use of 4% PFA in CSB for experiments. Images were taken at 60X with no additional post-processing.

Figure S3. Image analysis pipeline and classification of mitochondria. We began analyzing images of mitochondria by creating Z-stacks of individual cells. These Z-stacks were then compiled into a single image via a 3-D compression algorithm. Background subtraction was then conducted on the resulting image, followed by filtering with an FFT band-pass filter and finally thresholding by an adaptive thresholding algorithm. Random forest was finally applied as described to create a fully phenotyped cell.

Figure S4. Schematic of random forest classification of mitochondrial morphology. Following image thresholding, individual mitochondrial fragments and various shape parameters for fragments are identified. These shape parameters are then fed into a pre-trained (via manual mitochondria classification) random forest classifier where statistical voting results in a classification for the region of either punctate, intermediate or filamentous. Classifications for each mitochondrial region are then returned to the original image so that whole cell analysis of mitochondrial distributions can be conducted.

Figure S5. In silico mitochondrial classification. To test the accuracy of the trained random forest classifier, we manually created example sets of mitochondria of known phenotype (top panel, from left to right, punctate, intermediate, and filamentous mitochondria). These example sets were then run through the classification algorithm as stand-alone sets (that is, only the punctate, then only the intermediate etc.) as well as in a single combined set. The bottom panel shows the computer classification for the simulated mitochondria, where green corresponds to mitochondria classified as punctate, pink as intermediate, and blue as filamentous. In all cases the algorithm was accurate in classifying the example sets.

Figure S6. Verification of segmentation. (A) Individual OVCA-429 tumor cells were identified by user input and then automatically segmented. Shown is a random selection of 32 cells from the same population. Scale bar represents 10 µm. (B) OVCA-429 tumor cell populations were imaged either before treatment or following treatment with agents known to cause fragmentation and segmented and classified. The results were consistent with expected outcomes for Antimycin A and FCCP.

Figure S7. Single cell distribution of mitochondria for adherent cell lines. Distributions of mitochondria are presented as single cells, where the Y-axis represents each cell and the X-axis is the percentage of the total number of mitochondria.

Figure S8. Population distribution of mitochondria. (A) Box plot of mitochondrial distribution, where the box represents the 25-75th percentile distribution of populations, and whiskers represent 10-90th percentile distribution for all cultured cell lines. (B) The total mitochondrial area and (C) density are also shown, where each dot represents a single cell, black bars represent the population median and red whiskers represent 25-75th percentiles.

Figure S9. Scatter plot representation of mitochondrial distributions. Cultured cell lines (left panel) and patient derived xenograft samples (right panel) are shown in space where each corner of the triangle represents a pure population of fragmented, filamentous, or intermediate mitochondria. Single cells for each population are plotted in space in proportion to their mitochondrial morphological makeup. Each dot represents a single cell of the described population.

Figure S10. Mitochondrial protein level analysis in patient cells. Lysates from patient derived xenografts were stained for Drp1, Mfn1, Mfn2, & Opa1 with GAPDH as a normalization control. (A) Representative Western blots for each protein. (B) Quantified protein amounts normalized to GAPDH were plotted against the percentage of punctate mitochondria (an avatar for overall mitochondrial morphology) and correlation analysis was conducted showing limited relationships between individual proteins and punctate mitochondria.

Figure S11. In vitro cell response to cisplatin treatment. (A) OVCA-429 cells were analyzed either without or 72 hours after treatment with 10 µM cisplatin. Note the considerable reduction in filamentous mitochondria as well as the increase in total mitochondrial area (B). (C) Dose response curve of cisplatin plotting cell viability (solid red line, right axis) or the change in mitochondrial morphology (dashed green line, left axis) with treatment in OVCA-429 cells, as well as A2780 (D), A2780-Pt-Res (E) and Patient H (F) cell lines. In all cases, mitochondrial changes occur earlier and in lower doses compared to cell viability measurements. (G) Representative images of control (left panel) and treated (right panel) OVCA-429 cells. Scale bars represent 5 µm.

Figure S12. Fine needle aspirate in mouse tumors to assess mitochondrial change following cisplatin treatment (10 mg/kg). Mice 1-3 (M1-3) have cisplatin-sensitive A2780 tumors while mice 4-6 (M4-6) have cisplatin-resistant tumors. All animals were biopsied

before (pre-tx) and 36 hours following (post-tx) cisplatin treatment. Note the shift of filamentous mitochondria in responders, whereas few changes occur in non-responders. Each graph is the composite of 50-100 cells.

Figure S1

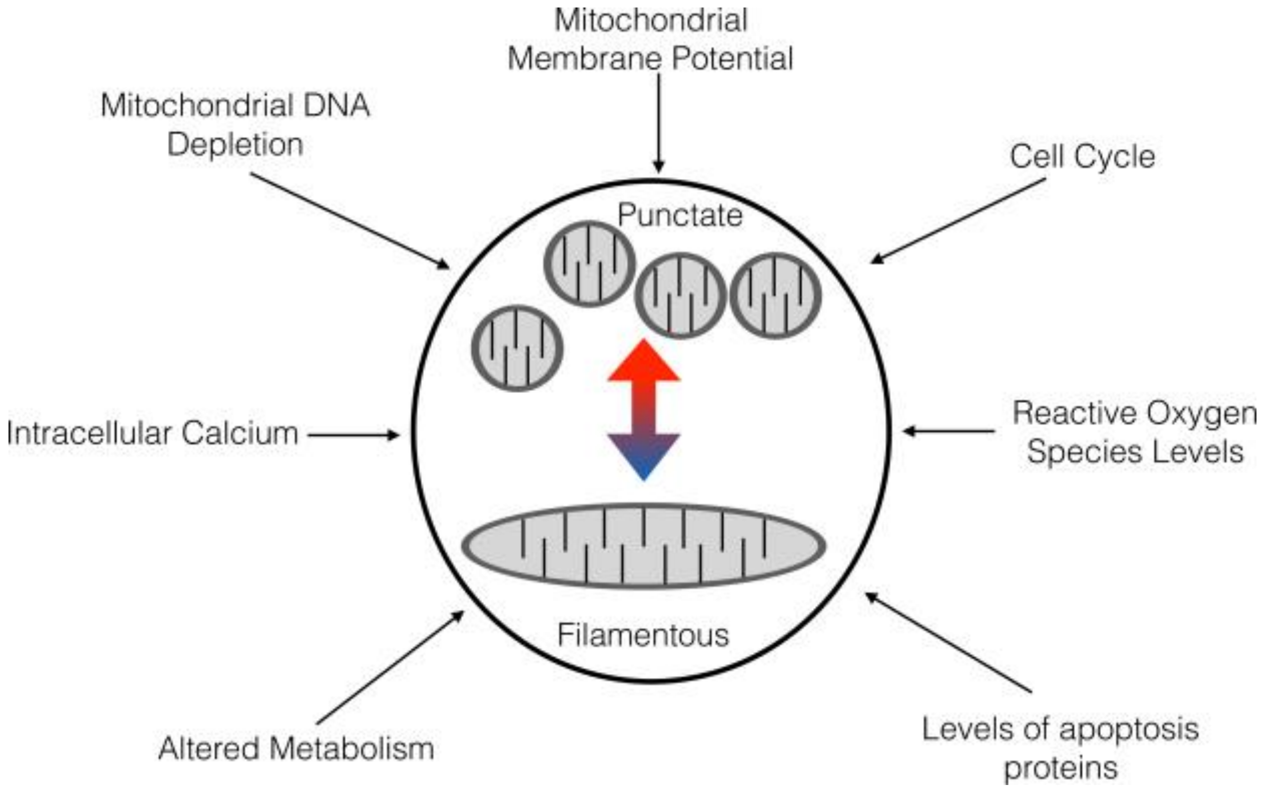
Mitochondrial
Membrane Potential
Mitochondrial DNA
Depletion
Cell Cycle
Punctate
Intracellular Calcium
Reactive Oxygen
Species Levels
Filamentous
Altered Metabolism
Levels of apoptosis
proteins

Figure S2

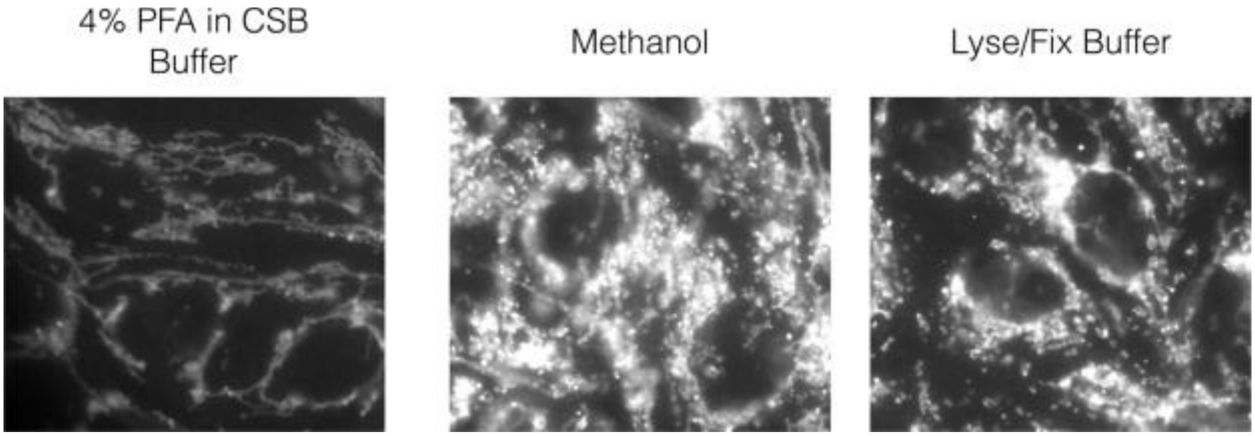

Figure S3

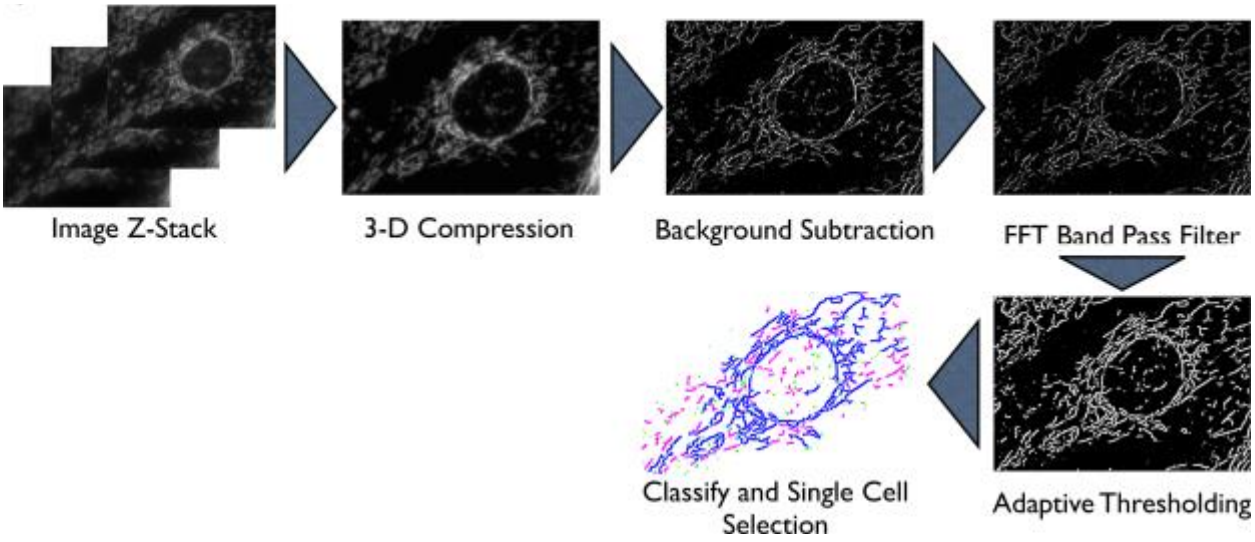
Image Z-Stack
3-D Compression
Background Subtraction
FFT Band Pass Filter
Adaptive Thresholding
Classify and Single Cell Selection

Figure S4

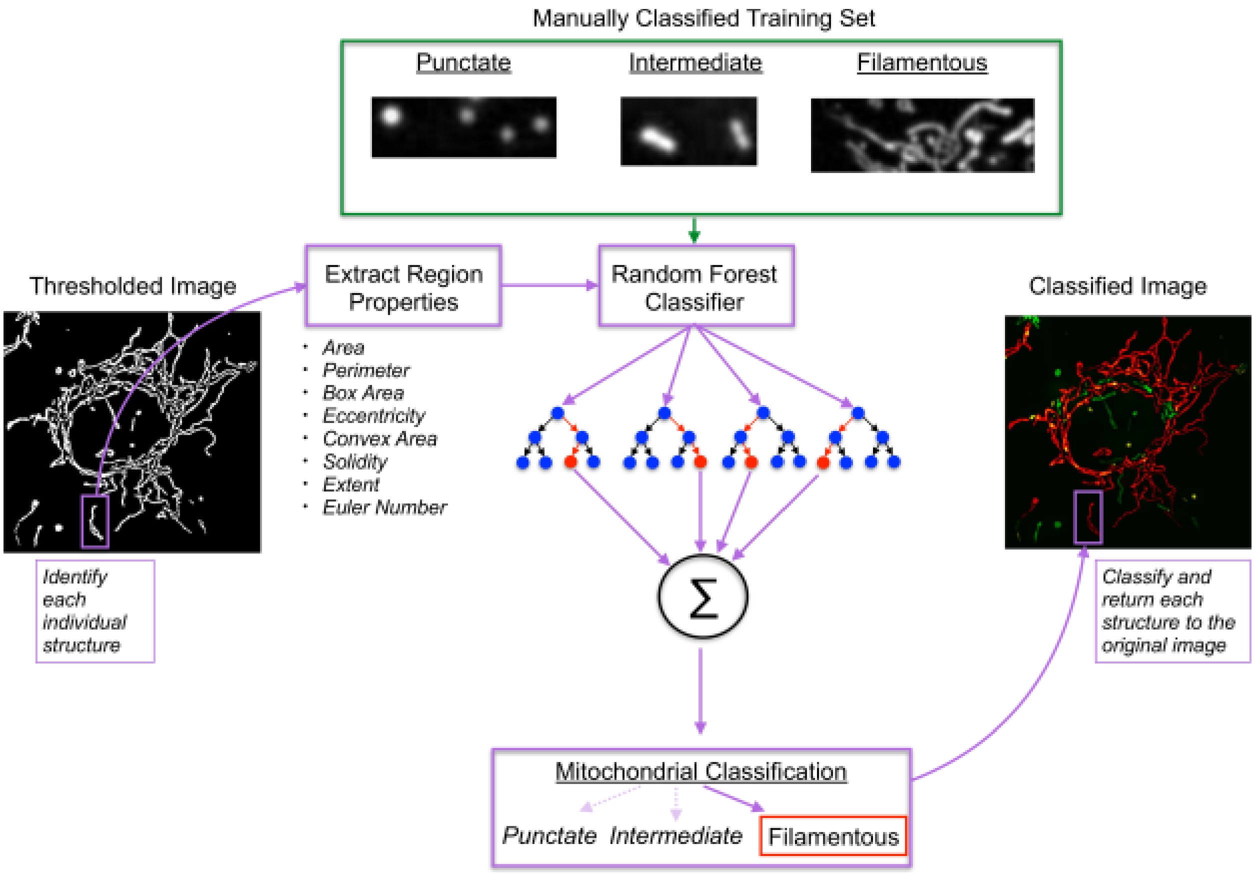

Manually Classified Training Set
Punctate
Intermediate
Filamentous
Thresholded Image
Extract Region Properties
Random Forest Classifier
Classified Image
Area
Perimeter
Box Area
Eccentricity
Convex Area
Solidity
Extent
Euler Number
Σ
Identify each individual structure
Classify and return each structure to the original image
Mitochondrial Classification
Punctate
Intermediate
Filamentous

Figure S5

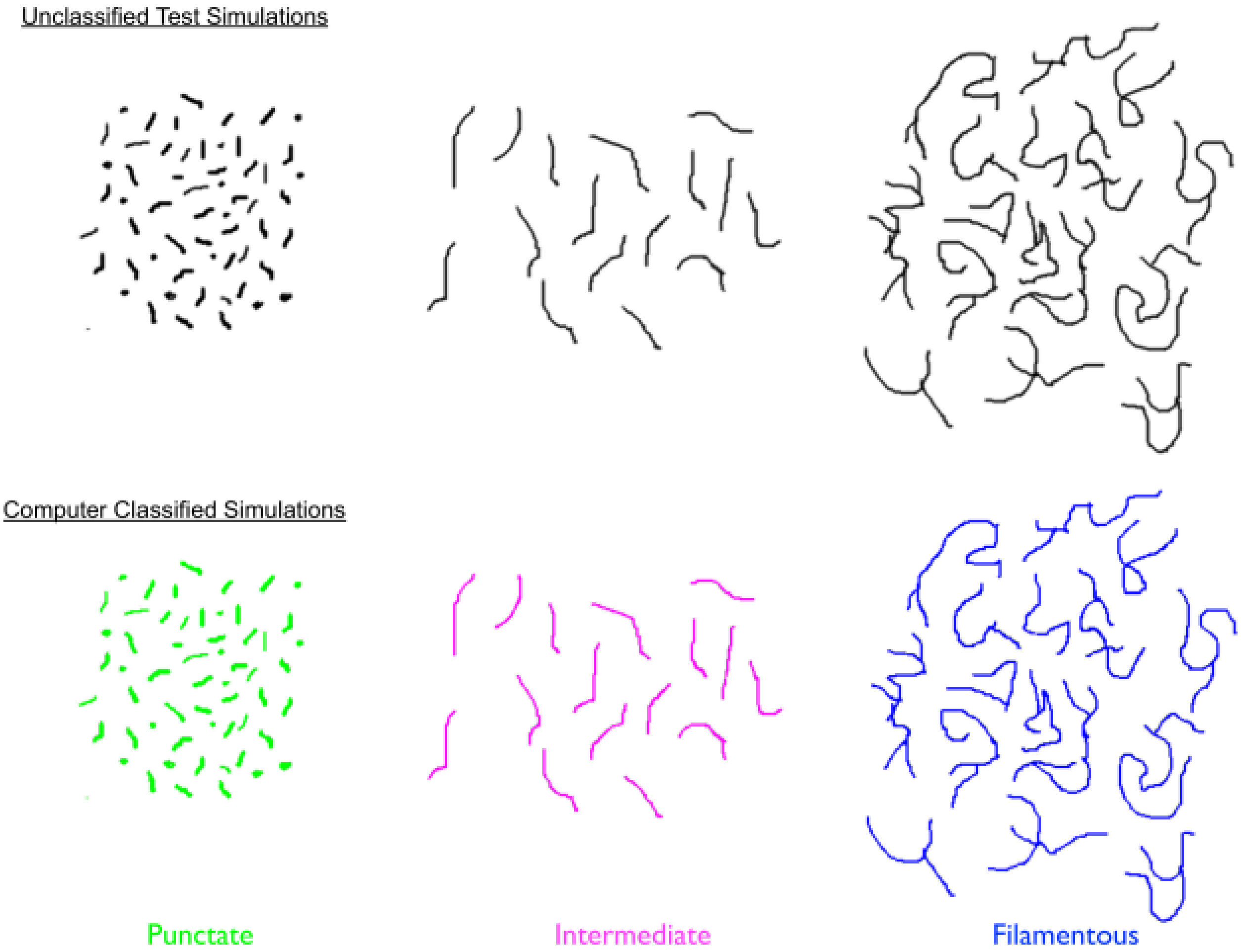

Unclassified Test Simulations
Computer Classified Simulations
Punctate
Intermediate
Filamentous

Figure S6

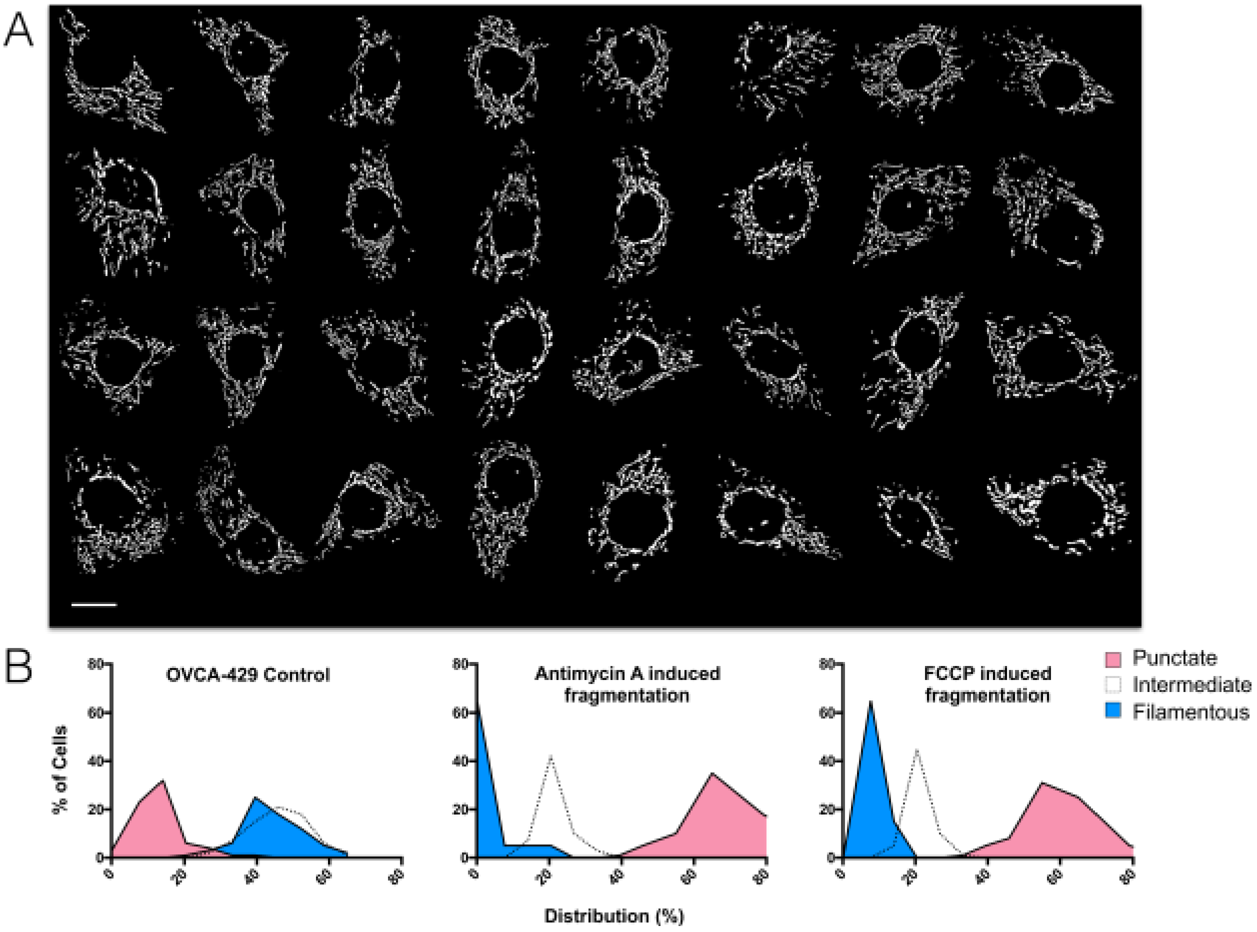

A
B
OVCA-429 Control
Antimycin A induced fragmentation
FCCP induced fragmentation
% of Cells
Distribution (%)
Punctate
Intermediate
Filamentous

Figure S7

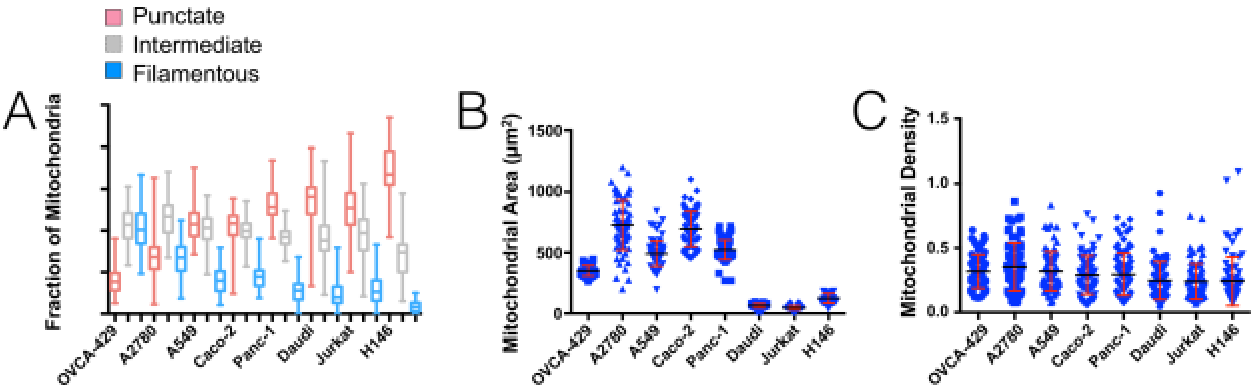

Punctate
Intermediate
Filamentous
A
Fraction of Mitochondria
OVCA-429
A2780
A549
Caco-2
Panc-1
Daudi
Jurkat
H146
B
Mitochondrial Area (µm²)
1500
1000
500
0
C
Mitochondrial Density
1.5
1.0
0.5
0.0

Figure S8

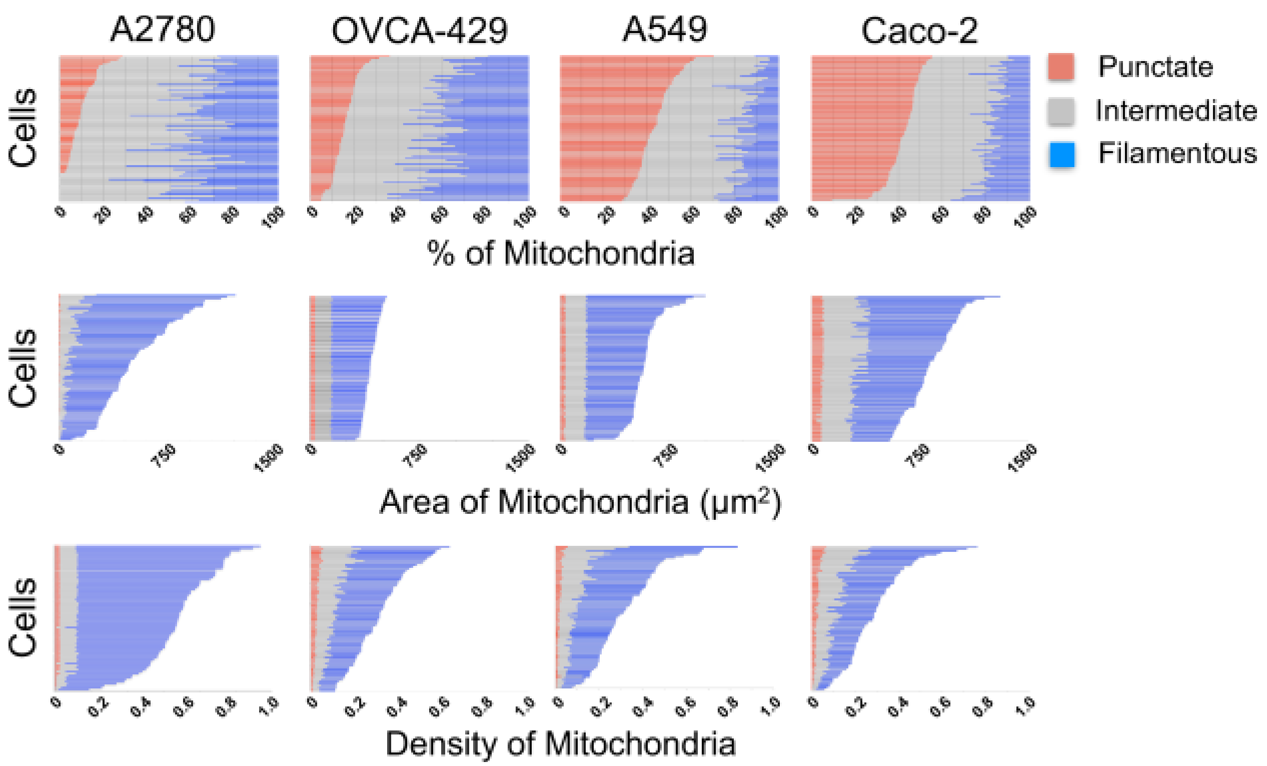
A2780
OVCA-429
A549
Caco-2
Punctate
Intermediate
Filamentous
Cells
0 20 40 60 80 100
% of Mitochondria
Cells
0 750 1500
Area of Mitochondria (μm²)
Cells
0 0.2 0.4 0.6 0.8 1.0
Density of Mitochondria

Figure S9

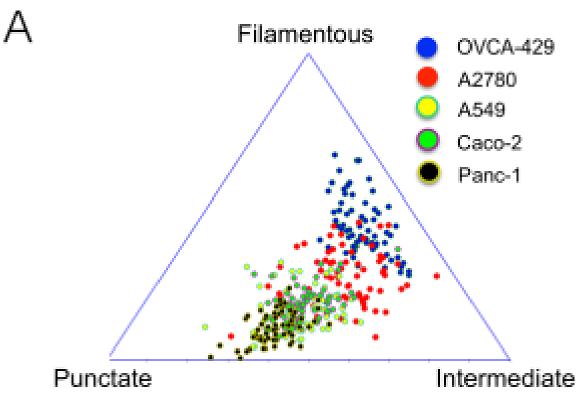
A
Filamentous
OVCA-429
A2780
A549
Caco-2
Panc-1
Punctate
Intermediate
B
Filamentous
Pt Ab
Pt D
Pt AF
Pt G
Pt H
Punctate
Intermediate

Figure S10

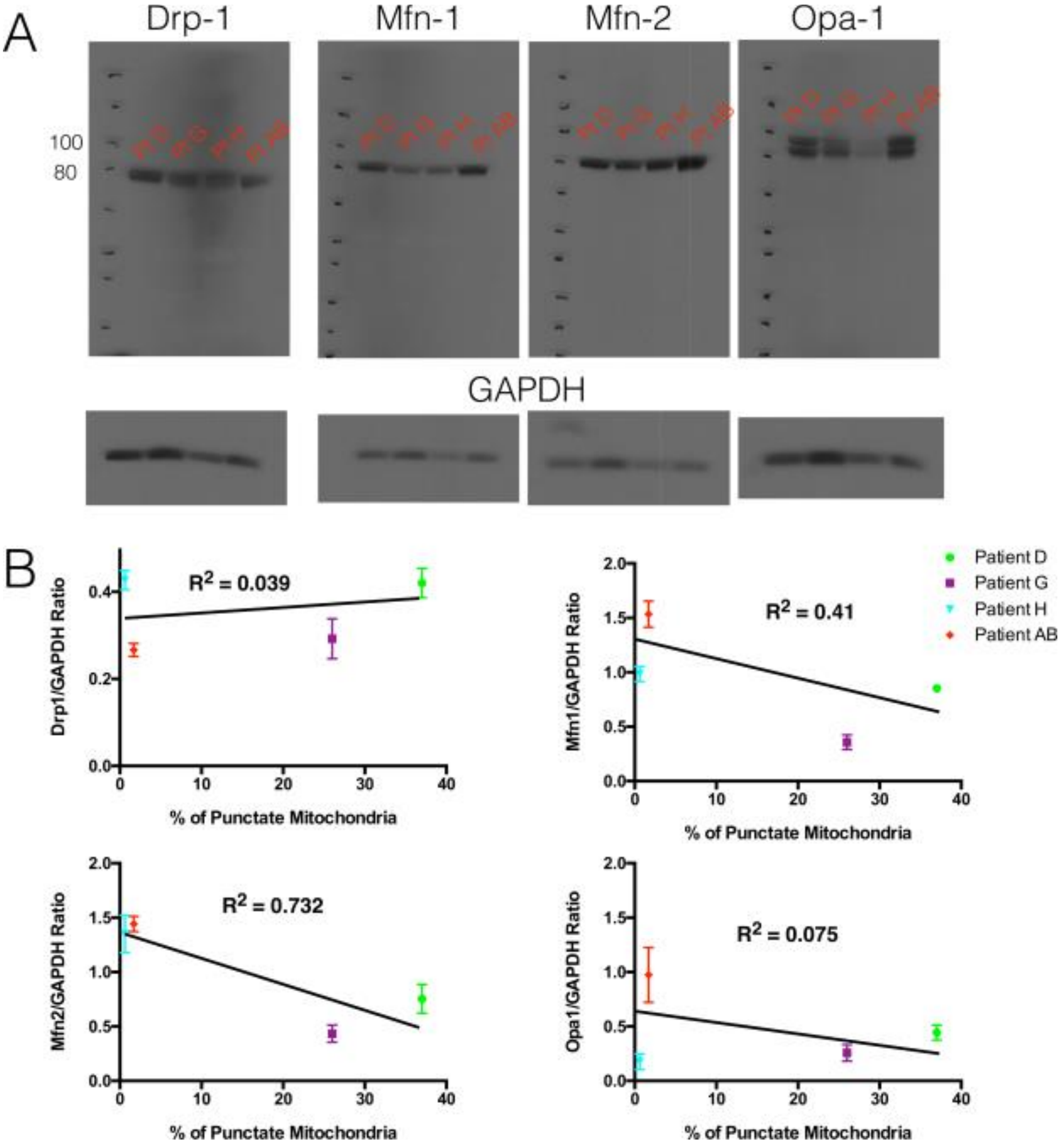
A
Drp-1
Mfn-1
Mfn-2
Opa-1
100
80
GAPDH
B
R2 = 0.039
Drp1/GAPDH Ratio
% of Punctate Mitochondria
R2 = 0.41
Mfn1/GAPDH Ratio
Patient D
Patient G
Patient H
Patient AB
R2 = 0.732
Mfn2/GAPDH Ratio
R2 = 0.075
Opa1/GAPDH Ratio

Figure S11

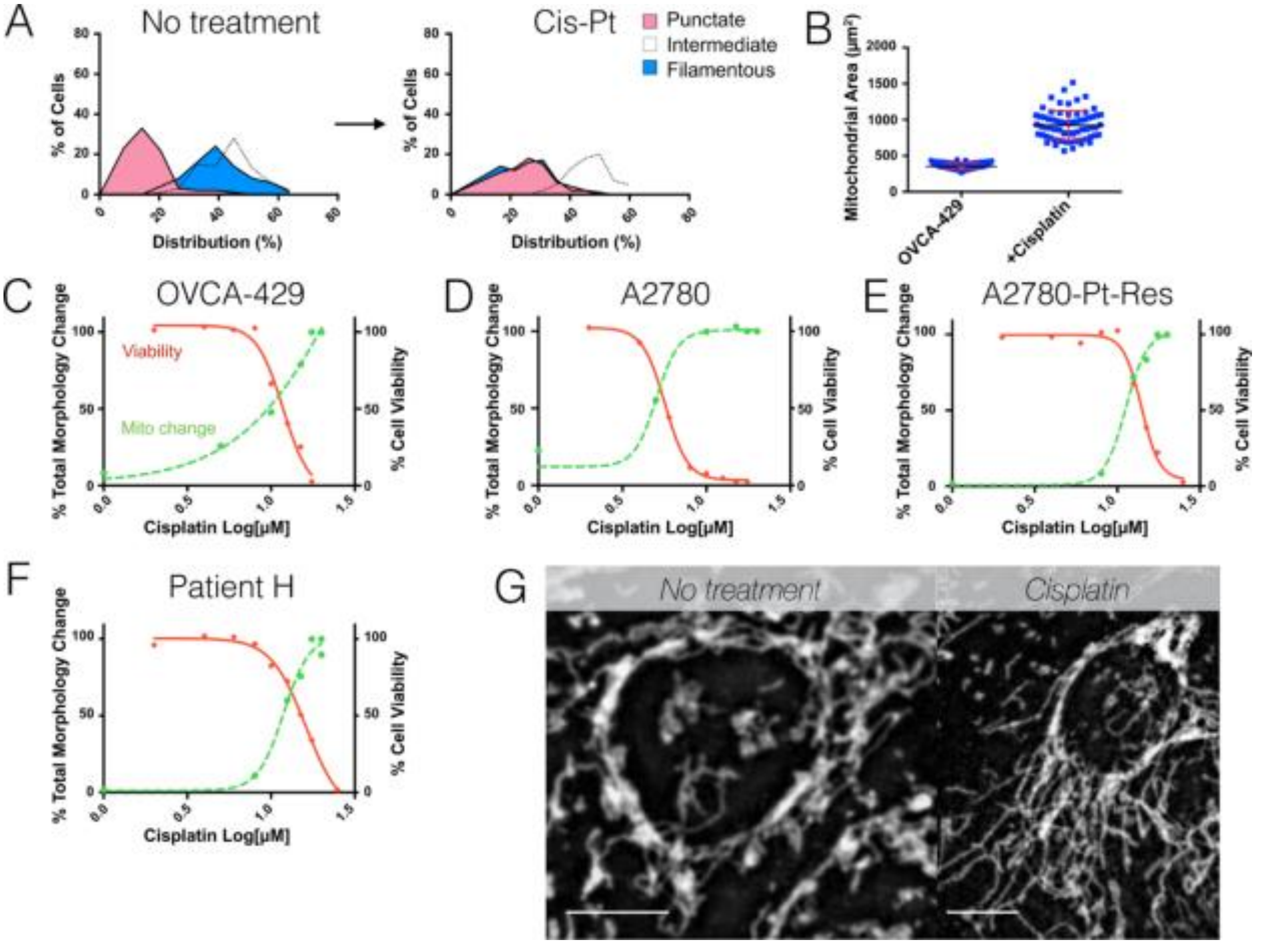

A
No treatment
Cis-Pt
Punctate
Intermediate
Filamentous
% of Cells
Distribution (%)
B
Mitochondrial Area (μm²)
OVCA-429
+Cisplatin
C
OVCA-429
Viability
Mito change
D
A2780
E
A2780-Pt-Res
F
Patient H
% Total Morphology Change
% Cell Viability
Cisplatin Log[μM]
G
No treatment
Cisplatin

Figure S12

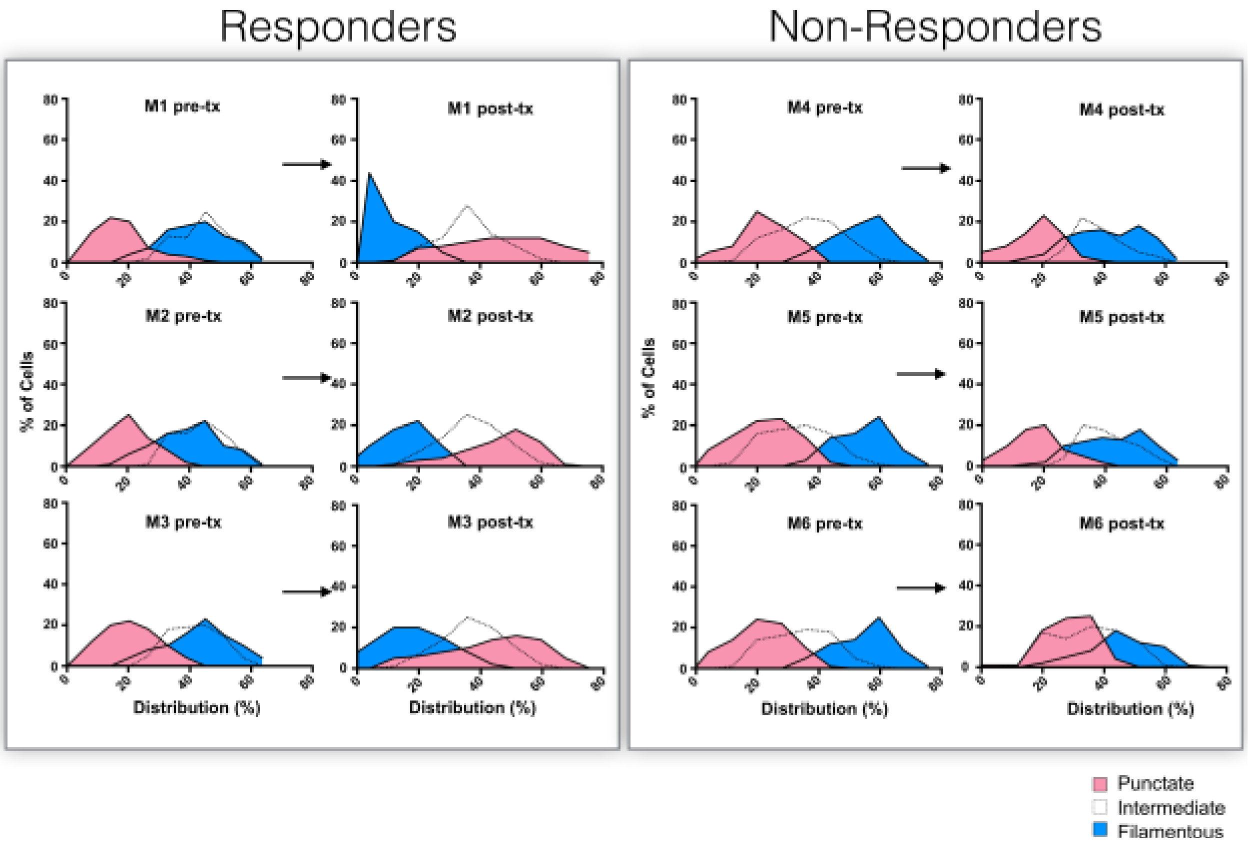
Responders
Non-Responders
M1 pre-tx
M1 post-tx
M2 pre-tx
M2 post-tx
M3 pre-tx
M3 post-tx
M4 pre-tx
M4 post-tx
M5 pre-tx
M5 post-tx
M6 pre-tx
M6 post-tx
% of Cells
Distribution (%)
Punctate
Intermediate
Filamentous
